# Noise estimation from averaged diffusion weighted images: Can unbiased quantitative decay parameters assist cancer evaluation?

**DOI:** 10.1002/mrm.24877

**Published:** 2013-08-01

**Authors:** Nikolaos Dikaios, Shonit Punwani, Valentin Hamy, Pierpaolo Purpura, Scott Rice, Martin Forster, Ruheena Mendes, Stuart Taylor, David Atkinson

**Affiliations:** Centre for Medical Image Computing, Division Medical Physics and Bioengineering, University College LondonLondon, UK; Centre for Medical Imaging, Division of Medicine, University College LondonLondon, UK; Department of Head and Neck Oncology, University College London HospitalLondon, UK

**Keywords:** diffusion weighted magnetic resonance imaging, noise estimation, IVIM

## Abstract

**Purpose:**

Multiexponential decay parameters are estimated from diffusion-weighted-imaging that generally have inherently low signal-to-noise ratio and non-normal noise distributions, especially at high *b*-values. Conventional nonlinear regression algorithms assume normally distributed noise, introducing bias into the calculated decay parameters and potentially affecting their ability to classify tumors. This study aims to accurately estimate noise of averaged diffusion-weighted-imaging, to correct the noise induced bias, and to assess the effect upon cancer classification.

**Methods:**

A new adaptation of the median-absolute-deviation technique in the wavelet-domain, using a closed form approximation of convolved probability-distribution-functions, is proposed to estimate noise. Nonlinear regression algorithms that account for the underlying noise (maximum probability) fit the biexponential/stretched exponential decay models to the diffusion-weighted signal. A logistic-regression model was built from the decay parameters to discriminate benign from metastatic neck lymph nodes in 40 patients.

**Results:**

The adapted median-absolute-deviation method accurately predicted the noise of simulated (*R*^2^ = 0.96) and neck diffusion-weighted-imaging (averaged once or four times). Maximum probability recovers the true apparent-diffusion-coefficient of the simulated data better than nonlinear regression (up to 40%), whereas no apparent differences were found for the other decay parameters.

**Conclusions:**

Perfusion-related parameters were best at cancer classification. Noise-corrected decay parameters did not significantly improve classification for the clinical data set though simulations show benefit for lower signal-to-noise ratio acquisitions.

Diffusion-weighted (DW) images have an inherently low signal-to-noise ratio (SNR) owing to the diffusion weighting and long echo times. The magnitude images exhibit a non-normal noise distribution, especially when employing high *b*-values where the SNR is worse 1. Intravoxel incoherent motion theory predicts that DW signal will be attenuated by both tissue perfusion and diffusion 2 components, resulting in a drop of signals at the lower *b*-values that is more rapid than predicted by a monoexponential (ME) model 3. Mathematical models, such as the biexponential (BE) 2 and stretched exponential (SE) 4, have been proposed to describe this nonME behavior, and are often fitted to DW images using nonlinear regression (NR) algorithms. An overview of these mathematical models is given in the Theory section. Conventional NR algorithms assume a normal noise distribution and will induce a bias in the estimated signal decay parameters. Specifically, it has been shown 5,6 that conventional NR algorithms can result in an underestimation of the apparent-diffusion-coefficient (ADC). Accurate evaluation of unbiased signal decay parameters is essential for staging, prognostication, and classification of tumor tissue.

To correct the noise-induced bias, it is necessary to know the expected probability distribution function (pdf) of the noise in the images used to calculate parameters. The noise in the underlying complex data is normally distributed but the magnitude operation (to reduce the effect of motion-induced phase shifts), image averaging, and the reconstruction technique can all lead to non-normal noise distributions. The calculation of magnitude images from the real and imaginary components of data with normally distributed noise results in images with Rician-distributed noise 7,8. If multiple receiver coils have been used during the acquisition, noise follows a noncentral-*χ* or Rayleigh distribution, depending on the reconstruction 1,9. Furthermore, this noise is dependent on coil sensitivities and varies spatially within the image. In diffusion-weighted-imaging (DWI), usually three gradient directions are applied and the trace of the diffusion tensor is calculated to form isotropically weighted images; the noise of trace DW images has been shown to follow Rician-distribution 10. If DW images are averaged, then according to the central limit theorem the average of a sufficient number of independent identically distributed measurements (with a finite mean and variance of their pdf) approaches a Gaussian distribution. Estimation of the noise distribution is of interest and to accurately estimate noise, Donoho 11 proposed the median-absolute-deviation (MAD) estimator in the wavelet domain for Gaussian noise, which was subsequently adapted for Rician noise 12,13.

The aim of this article is to correct the noise-induced bias of the signal decay parameters and to determine whether noise-corrected quantitative parameters are improved classifiers of tumor tissue. Focus is given to averaged trace DW images because in clinical practice DW images are averaged to improve the SNR. A closed form approximation pdf is proposed that can accurately approximate the noise distribution of averaged trace DW images and is incorporated within the MAD estimator. The proposed adapted MAD method is able to estimate noise from averaged and nonaveraged magnitude images.

Two NR algorithms (median [MD] and maximum probability [MP]) that account for the underlying noise 14 were implemented to provide unbiased signal decay parameters. The impact of the MD and MP algorithms versus a conventional NR algorithm was assessed for ME, BE, and SE models. A summary of all the abbreviations used throughout this article is provided in Table1.

**Table 1:** Synopsis of the Signal Decay Parameters per Mathematical Model and the Curve-Fitting Algorithms

Decay models	ADC	Pseudo-ADC	Perfusion fraction	Heterogeneity index
ME	*D*_ME_			
ME for *b* = 0, 50, and 100 s/mm^2^	*D_f_*_ast_			
ME for *b* = 300, 600, and 1000 s/mm^2^	*D*_slow_			
BE	*D*_BE_	D^*^_BE_	*f*	
SE	*D*_SE_			*α*
*Nonlinear regression fitting algorithms*				
Dantzig's simplex algorithm to minimize the l1-norm between:				
Decay model to measured signal (Eq. [6])		NR		
Maximum probability value of the pdf^a^ to measured signal (Eq. [7])		MP		
Median value of the pdf^a^ to measured signal (Eq. [8])		MD		

a Expected probability distribution function of the measured signal, *p* (measured signal decay model).

The performance of the proposed adapted MAD noise estimator and the impact of accounting for the underlying noise were evaluated using simulated DW images and data from two subjects imaged at different SNRs including noise-only reconstructions.

The clinical impact of accounting for the underlying noise of trace DW images and fitting the DW signal decay with a non-ME decay model was assessed in 24 patients with histologically confirmed head and neck squamous cell carcinoma cervical nodal metastases, and 16 normal volunteers. Decay models were fitted to the measured DW signal with a conventional NR algorithm and with an MP algorithm that accounts for the underlying noise. The ability of the estimated decay parameters to classify nodes on patients with known head and neck squamous cell carcinoma was assessed using a logistic regression analysis.

## THEORY

### Mathematical *Models*

Different mathematical models have been suggested that describe the non-ME diffusion signal decay and the two examined in this study are the SE 4 and BE 2 models because they provide simple direct physiological interpretations of the involved parameters 15. Including a ME model as a reference, the models used here are described below.

#### Monoexponential model


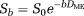
1where *S_b_* and *S*_0_ are the signal intensities with diffusion weights *b* and *b* = 0 s/mm^2^, respectively, and *D*_ME_ is the estimated ADC.

ADC maps derived exclusively from low *b*-values (<200 s/mm^2^) are sensitive to the perfusion effect, whereas those from late *b*-values (>200 s/mm^2^) are insensitive to the perfusion effect 15.

#### Biexponential model


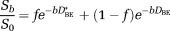
2where *D*_BE_ is the ADC attributed to diffusion, D*_BE_ is the pseudo-diffusion component that accounts for the perfusion effect and *f* ϵ [0, 1] is the perfusion fraction. The value of D*_BE_ is associated with the microcirculation of blood in the capillaries and is greater than *D*_BE_, which is associated with the diffusion of water molecules 2. For low *b*-values, the perfusion effect can govern the decay, whereas for higher *b*-values the perfusion contribution is near zero.

#### Stretched exponential model


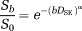
3where *D*_SE_ is the estimated ADC and *α* ***ϵ*** (0, 1] is the heterogeneity index. For *α* = 1, the SE is equivalent to the ME decay, and for *α* close to zero it resembles a high degree multiexponential decay.

### Curve Fitting Algorithms

To correct for the bias induced when data follow a non-normal distribution, the measured signal can be fitted with an NR algorithm to the “center” of the expected pdf. The center of the expected pdf can be attributed to the mean, median, or MP value. In clinical practice, the number of samples is limited and hence the median or MP schemes are preferred.

The median value (MD) of the pdf of the measurements *M_b_* with standard deviation *σ* of the noise corrupting the real and the imaginary data can be calculated numerically from the following equation 
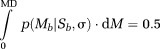
4

The MP value of a pdf is calculated from the following equation 
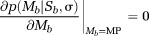
5

A fitting algorithm was used to find the decay parameters that minimize the l1-norm between the measured data and the:

1. i Predicted decay signal, *L*_NR_ (NR, Eq. [6]).
2. ii Maximum probability, *L*_MP_ (MP, Eq. [7]).
3. iii Median value, *L*_MD_ (MD, Eq. [8]).

These parameters are summarized in Table1. Parameter fitting can be affected by noise, outliers, and the presence of local minima in the cost function. We chose to use a Simplex algorithm (fminsearch in MATLAB (The Mathworks Inc., Natick, MA) and an l1-norm in the cost function to improve robustness 16. Specifically, the optimized likelihood functions are 
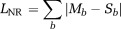
6

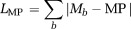
7

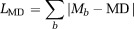
8

Maximum likelihood optimization algorithms that maximize a log likelihood function derived from the expected pdf have been proposed to provide unbiased ADC estimates. Kristoffersen 14 has shown that although maximum likelihood algorithms are possible for averaged data, they are time consuming and they do not perform better than the MD and the MP algorithms.

### Noise Distribution of Acquired DW Images

Table1 of Dietrich et al. 1 gives an overview of the expected noise distributions in magnitude MR images reconstructed with parallel imaging techniques. The noise-only acquisitions presented later in this study use the sensitivity encoding algorithm 17, and hence the expected noise distribution in the absence of averaging is Rician.

DW images for the patient population were reconstructed using the generalized autocalibrating partially parallel acquisition algorithm 18, where coil images are combined with the spatial-matched filter approach, and hence the expected noise distribution will again be Rician 1.

For all the acquired data in this article, the trace of the diffusion tensor was calculated, which has been shown to follow Rician distribution 10.

The pdf *p*_rice_ of the Rician distribution is given by 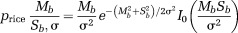
9where *σ* is the standard deviation of the noise corrupting the real and the imaginary data and *I*_0_ is the 0^th^ order modified Bessel function of the first kind.

If DW images are averaged but the number of averages is not sufficient to use a Gaussian distribution, the sum pdf, *p*_AV_, of independent and identically distributed random variables is given by the convolution of their pdfs. 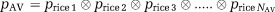
10where *N*_AV_ is the number of averages used. Convolution switches to multiplication after Fourier transformation 14, and hence the resulting pdf can be written as 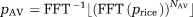
11

**FIG 1:**
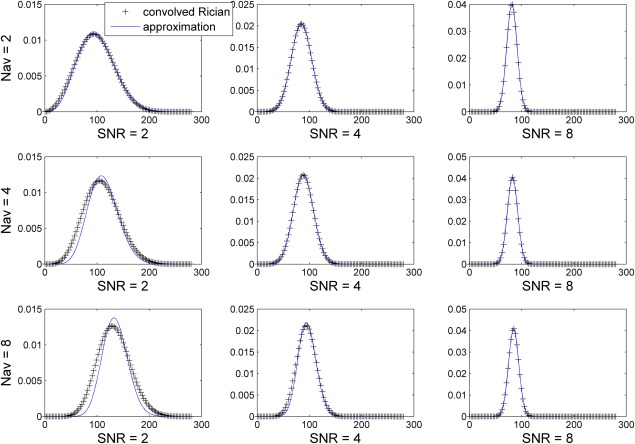
The probability density function of *N*_AV_-times convolved Rician distributions, and the closed form approximation (Eq.[12] for different SNR, and different number of averages *N*_AV_. [Color figure can be viewed in the online issue, which is available at wileyonlinelibrary.com.]

Hu 19 has suggested an approximation to the pdf of a Rician sum. A similar approximation is suggested in this project to approximate the pdf of averaged data 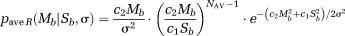


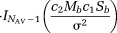
12where *c*_1_ and *c*_2_ are constants. To optimize the value of the constants *c*_1_, *c*_2_, Eq. [12] was fitted with an NR algorithm to Eq. [10]. Figure1 shows the suggested closed form approximation in Eq. [12], and the convolved Rician pdfs for different values of SNR, and numbers of averages.

### Noise Estimation

To estimate the noise from magnitude MR images, Coupé et al. 13 proposed an MAD technique adapted for Rician noise. In our study, the MAD estimator was further modified for the averaged Rice distribution. The 2D magnitude DW images were decomposed (Haar wavelet decomposition) into four sub-bands (LL, HL, LH, HH, L = low, and H = high frequencies). The lowest sub-band (LL) will mainly correspond to the object, and hence the LL sub-band is used to segment 20 the object from the background. Having segmented the object, the noise *σ*_G_ is estimated from the wavelet coefficients (*y_i_*) corresponding to its HH sub-band 11

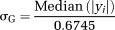
13

For low noise levels, Coupé's 13 suggestion to remove from the segmented object the areas with the highest gradient was followed. To derive an estimation of the variance *σ*_R_ for the expected noise distribution the iterative method suggested by Koay 12 was used. Initially, *σ* is set to *σ*_G_, and is updated using the formula 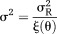
14where *ξ*(θ) is a correction factor based on the SNR θ = *S_b_*/*σ*. The correction factor *ξ*(θ) is calculated from the equation 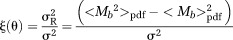
15where <*M_b_*> is the mean signal intensity and <*M_b_*^2^> is the second moment of the signal intensity. To calculate *ξ*(θ) a closed form of the expected pdf is needed.

The correction factor *ξ*(θ) for the approximation of the Rician sum pdf (Eq. [12] can be calculated using the substitutions *M*′*_b_* *=c*_2_*M_b_* and *S*′*_b_* *=c*_1_*S_b_*, where *c*_1_, *c*_2_ are the same constants as in Eq. [12]. The Rician pdf *p*_rice_(*M*′*_b_*|*S*′*_b_*,*σ*) is equal to the Rician sum pdf *p*_ave_*_R_*(*M_b_*|*S_b_*,*σ*), and hence the first and second moments of *M′_b_* are 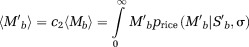


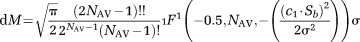


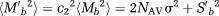


Through substitution of the first and second moment of *M*′*_b_* to Eq. [15], 
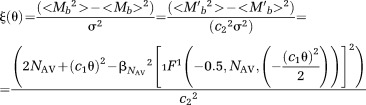
16where _1_*F*^1^ is the confluent hypergeometric function and *β*_NAV_ is a factor depending on the number of averages *N*_AV_ (analytic form given in Eq. [12]. For *N*_AV_ = 4, *β*_4_ = 35√*π*/16√2. SNR *θ* is updated through substitutions in Eq. [15] according to the equations, 
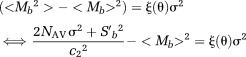


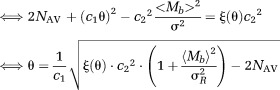
17where <*M_b_*> is the mean signal intensity. The *ξ*(θ) from the last iteration is used to calculate the unbiased estimate of the variance *σ* (Eq. [14]).

**FIG 2:**
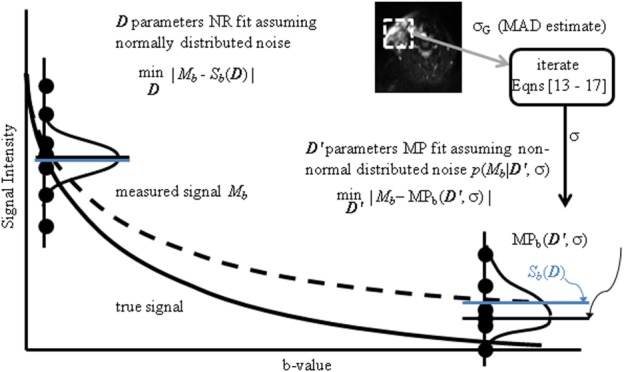
Illustration of parameter calculation. For a true signal (solid line), noise in magnitude images results in the measured signal *M_b_* (dashed line). Solid circles indicate the spread of measurements with their associated distribution. Conventional NR fits the modelled signal *S_b_* to *M_b_*, resulting in bias. Alternatively, signal decay parameters *D*′ are estimated by fitting the MP or the median (MD) value of the pdf of the underlying noise to *M_b_*. Inset image indicates the region used for the MAD estimate of the pdf. For clarity, only MP is shown in the figure. [Color figure can be viewed in the online issue, which is available at wileyonlinelibrary.com.]

An overview of the curve fitting algorithms and the noise estimator in the derivation of unbiased signal decay parameters is shown in Figure2.

## METHODS

### Simulated DW Images

Two nodes were randomly selected from the clinical data set: the first was drawn from patients with histologically positive neck nodal metastases from primary head and neck squamous cell carcinoma; the second from patients evaluated by MRI for mechanical causes of neck pain and no clinical/radiological suspicion of tumor. Median values of each DW image for each node were calculated. These values were used to generate noise-free DW 2D images at the six different diffusion weights b (0, 50, 100, 300, 600, and 1000 s/mm^2^) corresponding to the diffusion imaging protocol for clinical studies. The simulated images consisted of a circle with a homogenous signal intensity value equal to the median values of the benign node, and a smaller circle (within the previous one) with a homogenous value equal to the median value of the metastatic node.

Different Gaussian noise levels *σ*_l_ were applied to the real and imaginary part of the simulated DW images (*M_rb_*). The noisy magnitude DW images (*M_b_*) were then averaged four times (*N*_AV_ = 4), resulting in averaged magnitude DW images with applied noise *σ*_applied_ equal to 
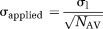
18

In *simulation* 1, the purpose was to estimate the applied noise *σ*_applied_ of a nonaveraged and an averaged DW image at b = 1000 s/mm^2^ with the proposed adapted MAD method.

In *simulation* 2, three different noise levels were applied, noise was estimated from the averaged DW images with the proposed adapted MAD method and incorporated into the MD and MP curve fitting algorithms to correct for the noise-induced bias. The three different noise levels correspond to SNR of ∼3, 5, and 8, respectively. The calculated signal decay parameters were estimated with the MD, MP, and conventional NR algorithms. The purpose of simulation 2 was to determine which algorithm provided decay parameters closest to the ground truth for different SNRs.

### Experimental Noise Scan

Axial DW images of the neck were acquired for two normal volunteers using a DWI spin-echo sequence on a Philips Achieva 3T MRI scanner with a 16-channel neurovascular receiver coil and reconstructed using the sensitivity encoding algorithm. Diffusion gradients were applied in three orthogonal directions at each of six *b*-values (0, 50, 100, 300, 600, and 1000 s/mm^2^). Images were reconstructed with a 240 × 240 matrix size and 0.95-mm in-plane pixel size for both subjects. One subject was acquired with 5-mm slice thickness, 6-mm gap, repetition time = 2 s, and echo time = 60 ms (*subject* 1). The other subject was acquired with reduced slice thickness to lower the SNR, specifically: 2.5-mm slice thickness, 3-mm slice gap, repetition time = 1.88 s, and echo time = 60 ms (*subject* 2). To measure noise after parallel imaging reconstruction, *b* = 1000 s/mm^2^ acquisition was repeated with the subject in the scanner and using the same receiver coils but no RF excitation so that only noise was received. The noise-only images were reconstructed using the same coil sensitivities and reconstruction parameters. This noise-only method was repeated for a scan with *N*_AV_ = 4. Noise was estimated with the proposed adapted MAD method (*σ_R_*) for the two subjects for *N*_AV_ = 1 and 4, for comparison, the noise was also estimated by fitting the expected pdf to the histogram from the noise-only data with an expectation maximization algorithm.

Sixteen single measurements for each of the six *b*-values were acquired for each subject. Sixteen signal decay parameters were calculated separately with the NR and the MP method from the 16 single measurements. Signal decay parameters were also estimated with the NR and MP methods from the averaged 16 DW images. An region of interest (ROI) was contoured on the cervical node and the median value of each signal decay parameter across the ROI was calculated.

### Patient Studies

Twenty-four consecutive patients (mean age, 58 years; standard deviation, 8 years; range, 43–79 years) satisfying inclusion criteria of histologically confirmed head and neck squamous cell carcinoma with unilateral cervical nodal metastatic disease at pretherapy staging, and 16 normal volunteers (mean age, 51 years; standard deviation, 14 years; range, 23–74 years) were recruited between March 2010 and June 2011. All patients underwent contrast enhanced neck-computed tomography, anatomical MRI, and ultrasound evaluation of the neck as part of routine clinical practice. Nodal status was confirmed by conventional imaging criteria (>0.6 cm short axis, round contour, irregular margins, necrosis, heterogeneous enhancement on CT/MRI) ± ultrasound guided fine needle aspiration (cytological sampling) of equivocal nodes. Volunteers had no previous history or current clinical/radiological suspicion of cancer and were recruited from a pool of patients undergoing neck MRI for mechanical causes of neck pain.

### DW Imaging

**FIG 3:**
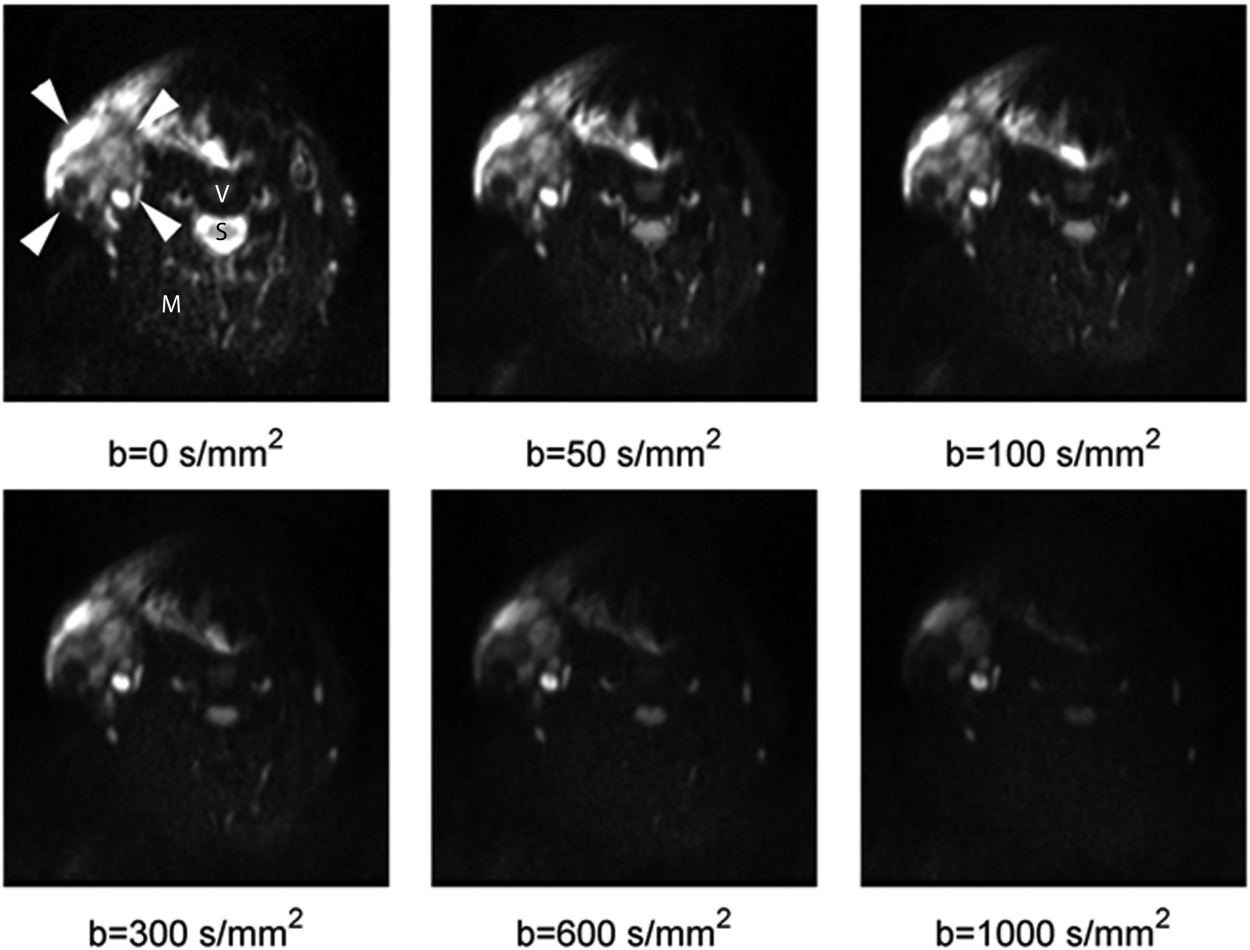
Echo planar images for different diffusion weightings of a slice with a metastatic lymph node for one of the 24 patients. Arrowheads on the *b* = 0 s/mm^2^ DW image show the position of the metastatic lymph node. Relevant anatomical landmarks were also annotated: S, spinal cord; V, vertebral body; and M, paravertebral muscles.

Axial DW images of the neck (base of skull to upper thorax) were acquired in the supine position using a short tau inversion recovery-echo planar imaging sequence on a 1.5T Siemens (Siemens, Erlangen, Germany) Avanto magnet with the manufacturer's carotid coils. Trace DW images of the head and neck were acquired with two receiver coils using generalized autocalibrating partially parallel acquisition. Coil images were combined using the spatial-matched filter approach provided as “adaptive combine.” Diffusion gradients were applied in three orthogonal directions at each of the six *b*-values (0, 50, 100, 300, 600, and 1000 s/mm^2^). The use of three successive orthogonal directions means that the ADC and mean diffusivity are rotationally invariant. Both DW images and *b* = 0 s/mm^2^ images were averaged four times to improve SNR (Fig.3. Images were acquired with a slice thickness of 4 mm, 0.4 mm of slice gap, and a matrix size of 128 × 128. Total acquisition time (of all six *b*-factors) for diffusion MR imaging was 6 min and 10 s. Sequence parameters are listed in Table2. Representative SNRs of the metastatic nodes were 20.3 (*b* = 0 s/mm^2^), 16.4 (*b* = 50 s/mm^2^), 14.1 (*b* = 100 s/mm^2^), 12.1 (*b* = 300 s/mm^2^), 10.9 (*b* = 600 s/mm^2^), and 9.7 (*b* = 1000 s/mm^2^). Likewise, representative SNRs of the benign nodes were 15.4 (*b* = 0 s/mm^2^), 9.6 (*b* = 50 s/mm^2^), 8.9 (*b* = 100 s/mm^2^), 7.2 (*b* = 300 s/mm^2^), 6.9 (*b* = 600 s/mm^2^), and 6.4 (*b* = 1000 s/mm^2^).

**Table 2:** Diffusion MR Sequence Parameters for Patient Scans on a Siemens Avanto 1.5T^a^

Parameter	Axial short tau inversion recovery-echo planar imaging DWI
No. of sections	42
Stacks	1
Field of view (cm)	20
Repetition time (ms)	8700
Echo time (ms)	88
Inversion time (ms)	180
Matrix	128 × 128
Section thickness (mm)	4
Section gap (mm)	0.4
Averages	4
Parallel acquisition	2
*b*-Values (s/mm^2^)	0, 50, 100, 300, 600 and 1000

a Siemens Avanto 1.5T has gradient field strength up to 45 mT/m and a slew rate up to 200 T/m/s.

### Derivation of Parametric Maps

Trace DW images were used to create decay parametric maps for each patient using both NR and MP algorithms separately.

ME ADC maps were generated using all six *b*-values (*D*_ME_), and fits of low (0, 50, and 100 s/mm^2^) and high (300, 600, and 1000 s/mm^2^) were also performed to separate the fast (*D*_fast_) and slow (*D*_slow_) diffusion components (Eq. [1]).

The BE model has three unknown parameters, which may cause the curve fitting algorithm to converge to a local minimum. Lemke et al. 21 reported that ideally more than 10 *b*-values are needed to perform reliably the BE model. To facilitate the convergence for the BE model, *D*_BE_ was initialized with the *D*_slow_ and D*_BE_ was initialized with the *D*_fast_ values. D*_BE_ and *f* maps were derived from the BE model (Eq. [2]. *D*_SE_ and *α* maps were derived using the SE model (Eq. [3]).

A synopsis of the signal decay parameters per mathematical model and the curve fitting algorithms is summarized in Table1.

### Image Analysis

For each patient, the single largest lymph node was contoured on the *b* = 300 s/mm^2^ images using Jim 5.0 software by two experienced radiologists in consensus and used for interrogation of parametric maps. Volumetric ROIs were drawn encompassing the benign or metastatic node while excluding any cystic component. A total of 40 individual nodes were sampled (one node per patient, 16 benign and 24 metastatic). The adapted MAD method was applied across a block (30 × 30 pixels) encompassing the ROI on each imaging slice.

Median of each volumetric ROI values for each node derived from both MP and NR estimated parametric maps were calculated.

### Statistical Analysis

Statistical analysis was performed using SPSS (SPSS Base 20.0 for Windows User's Guide. SPSS Inc., Chicago, IL).

A Mann–Whitney *U*-test (MWU sig) was performed to compare the median values of the signal decay parameters between metastatic and benign nodal groups. Models predictive of metastatic nodal status were derived using logistic regression. The Wilcoxon signed-rank test (W sig.) was used to compare decay parameters estimated with NR and MP. The ability of individual MP- and NR-derived signal decay parameters to predict metastatic nodal status was assessed by receiver operator characteristic (ROC) area under curve (AUC) analysis 22).

Separate multiparameter (mp) logistic regression models were built for NR and MP algorithms from their respective calculated signal decay parameters (mp-DWI NR, mp-DWI MP). Individual signal decay parameters that had significantly different (*P* < 0.05) median values between normal and metastatic nodes, higher AUC and that were uncorrelated from other parameters (Kendall tau close to zero) were included within each multiparametric model.

Leave-one-out analysis was used to assess the accuracy of predictive models on independent samples. One case (out of 40) was excluded, and a model generated from the remainder of the cases. The model was then tested on the excluded case and a predictive probability calculated, so that the excluded case is not used but is predicted. The process was repeated 40 times excluding successive cases in turn allowing calculation of 40 predictive probabilities. Leave-one-out analysis was implemented using SPSS syntax. A ROC was then created using the derived predictive probabilities.

A leave-two-out analysis was also implemented using SPSS syntax to further assess the accuracy of the predictive models on independent samples. Two cases (out of 40) were randomly excluded, and similarly to leave-one-out analysis a model was generated from the remainder of the cases. The process was repeated 1000 times randomly excluding two cases each time allowing calculation of 2000 predictive probabilities.

A ROC analysis was performed with SPSS for both leave-one-out and leave-two-out analysis using their derived predictive probabilities.

## RESULTS

### Simulated DW Images

**FIG 4:**
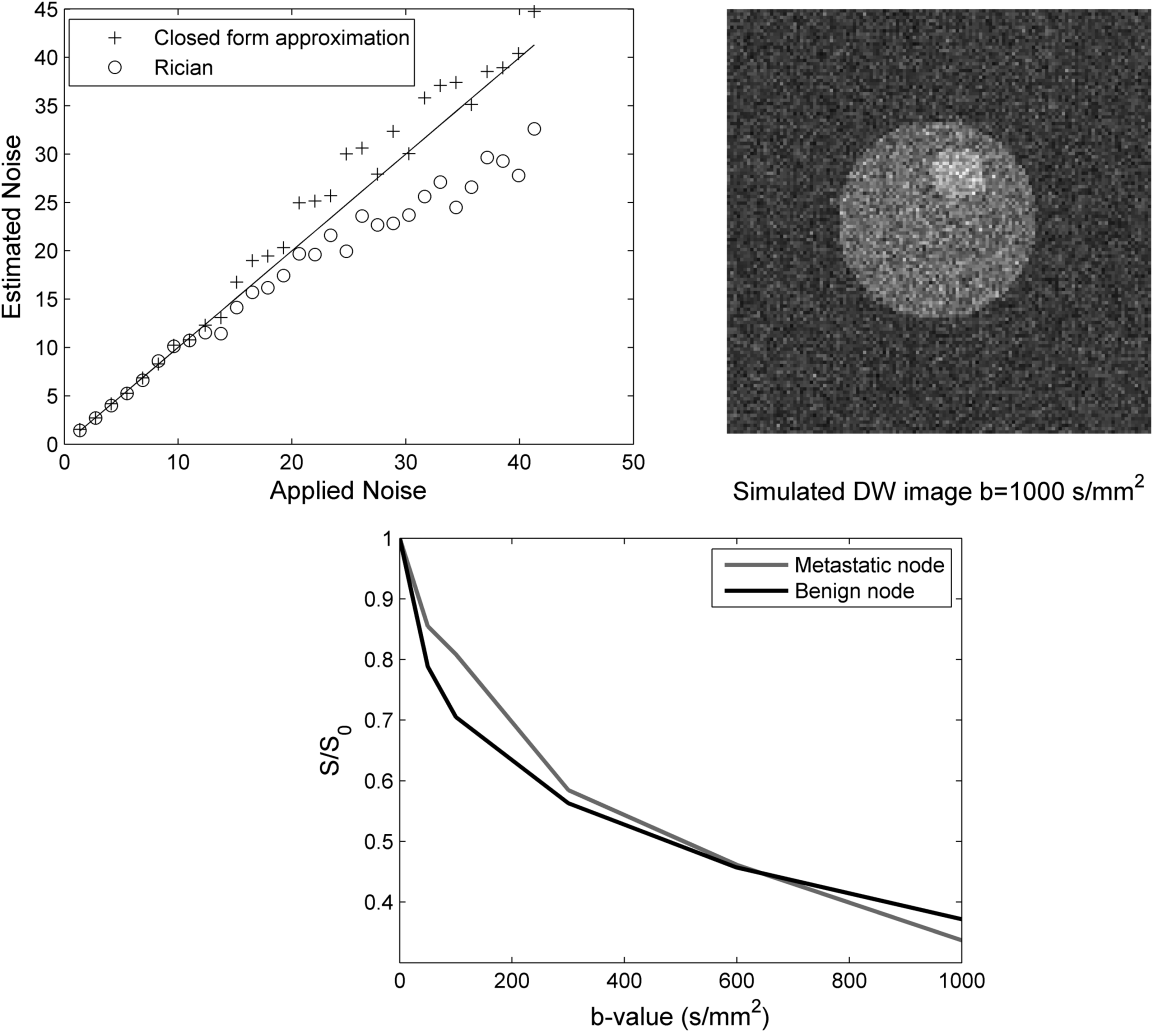
Estimated noise (top left) with the adapted median absolute deviation (MAD) method of averaged DW (*b* = 1000 s/mm^2^) images (top right) at different noise levels (*simulation* 1). The relative signal change *S*/*S*_0_ as a function of the *b*-value is shown (bottom) for the two areas that correspond to benign and metastatic nodes. The adapted MAD method using the closed form approximation (Eq. [12] predicted the applied noise with an ***R***^2^ = 0.96. If the adapted MAD method uses the less appropriate Rician distribution, ***R***^2^ = 0.65.

Figure4 (*simulation* 1) illustrates that the proposed adapted MAD method with the closed form approximation predicted the applied noise *σ*_applied_ of averaged (*N*_AV_ = 4) simulated DW *b* = 1000 s/mm^2^ images (with SNR values from 30 to 2) with an ***R***^2^ = 0.96. Although if the Rician distribution is used instead of the closed form approximation, the adapted MAD method 13 predicts the applied noise with an ***R***^2^ = 0.65. The proposed adaptation of the MAD method also predicted the applied noise of nonaveraged images (*N*_AV_ = 1) with an ***R***^2^ = 0.96, and the adapted Rician MAD method 13 predicts the applied noise with an ***R***^2^ = 0.95. Figure4 also shows the relative signal *S*/*S*_0_ change as a function of the *b*-value, where the larger circle corresponds to the median value of the benign nodes of the volunteer population and the inner circle corresponds to the median value of the metastatic nodes of the patient population.

*Simulation* 2 results for the averaged (*N*_AV_ = 4) DW images of the metastatic ROI are summarized in Table3. There is an advantage compensating for noise when the SNR is low, and this advantage diminishes at higher SNR values. For the ME model, the MP and MD algorithms recover the noise induced bias, whereas as expected NR algorithms underestimated the true *D*_ME_ up to 40% depending on the applied noise. For the SE and the BE model, the MP and MD algorithms are more accurate although the improvement is not as noticeable as for the ME model.

**Table 3:** Signal Decay Parameters Estimated from the Averaged Simulated DW Images at Different Applied Noise Levels *σ*_applied_ Using the MP, MD, and NR Algorithms (*simulation* 2)^a^

	Metastatic tissue						
	*D*_ME_	*D*_fast_	*D*_SE_	*α*	*D*_BE_	*f*	D^*^_BE_
TRUE	1.31	3.49	0.98	0.45	0.59	0.33	17.48
	SNR = 3						
NR	0.91	2.54	0.38	0.39	0.33	0.38	6.58
MP	1.24	3.48	0.66	0.43	0.49	0.35	10.99
MD	1.37	3.49	0.60	0.42	0.42	0.37	10.68
	SNR = 5						
NR	1.18	3.23	0.8	0.43	0.44	0.37	10.13
MP	1.3	3.51	0.96	0.45	0.56	0.34	13.59
MD	1.29	3.51	0.96	0.45	0.55	0.34	13.73
	SNR = 8						
NR	1.26	3.47	0.93	0.46	0.51	0.35	14.35
MP	1.3	3.47	0.95	0.46	0.59	0.33	16.39
MD	1.28	3.47	0.96	0.48	0.57	0.34	15.83

a The applied noise levels corresponding to SNR = 3, 5, and 8 of the *b* = 1000 s/mm^2^ DW image. The estimated decay parameters were compared with the ground truth decay parameters (true) derived from noise-free averaged DW images with the NR algorithm. Comparison includes the ROI corresponding to the metastatic tissue. Diffusion coefficients (abbreviations shown in Table1 are given in units of 10^−3^ mm^2^/s.

The MP-derived decay parameters are closer to the true decay parameters than the MD-derived decay parameters and hence MP was preferred for the clinical head and neck data.

### Experimental Noise Scan

**FIG 5:**
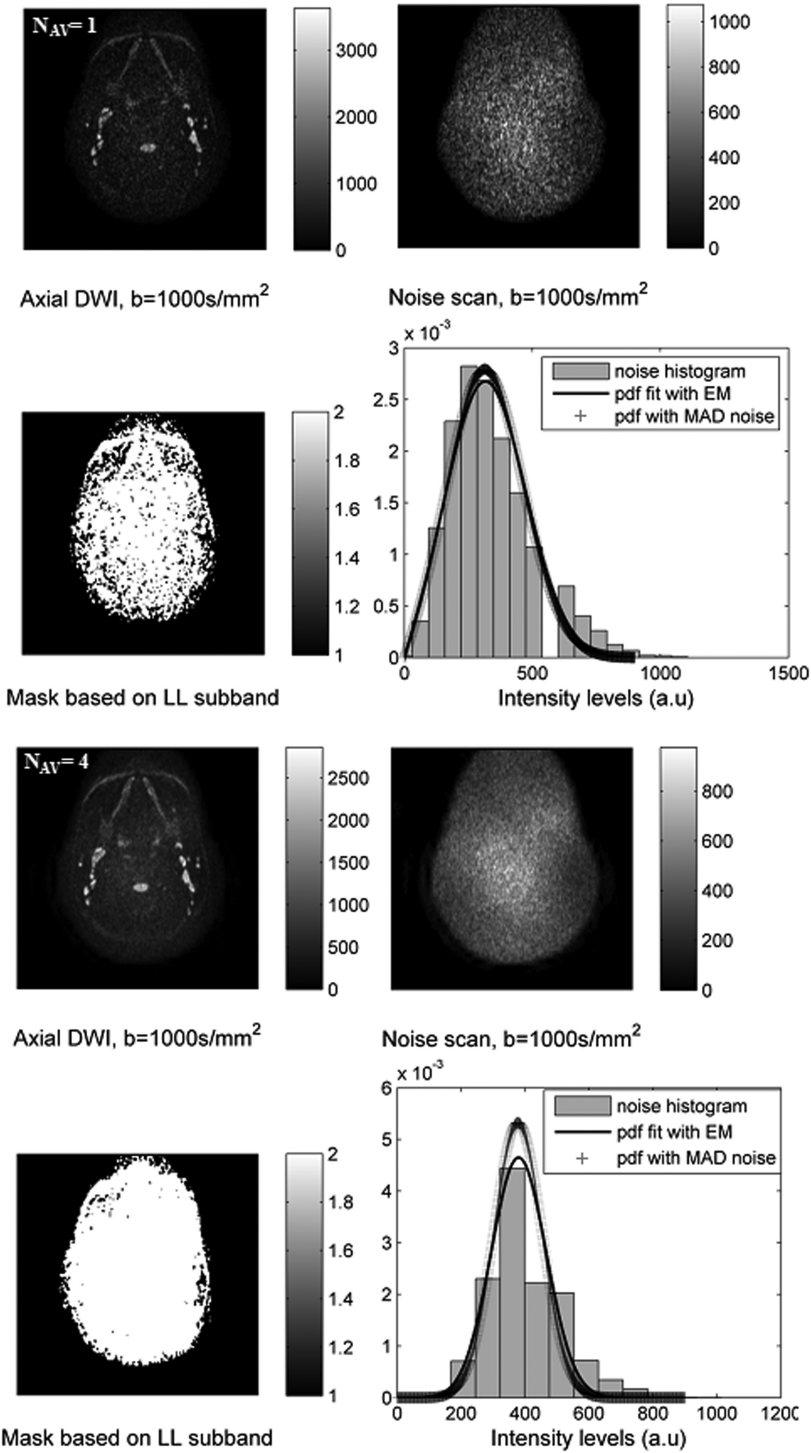
DW images (*b* = 1000 s/mm^2^) of *subject* 2 with *N*_AV_ = 1 (top two rows) and 4 (bottom two rows) at the position of the normal lymph node. Noise scans were acquired with no RF pulses. Masks of the subject corresponding to the LL sub-band estimated from the proposed adopted MAD method are also shown. The expected probability distribution function was fitted to the noise histograms (*N*_AV_ = 1 and 4) with expectation maximization for the noise-only scans, and with the MAD noise estimate on the conventional images.

A summary of the results for the two normal subjects is provided in Table4 and Figure5 shows histograms of the underlying noise, fitted with an expectation maximization algorithm and with the estimated noise from the proposed adapted MAD method for subject 2.

**Table 4:** Noise Estimates from the Neck DW Images with the Proposed Adapted MAD Method (*σ*_R_), and with the Expectation Maximization Fit (*σ*_T_) to the Underlying Noise Distribution Acquired Without RF Pulses^a^

	Slice thickness 5 mm	Slice thickness 2.5 mm
	*Subject 1*	*Subject 2*
*N*_AV_ = 1	*σ*_R_ = 45 (*σ*_T_ = 48)	*σ*_R_ = 154 (*σ*_T_ = 162)
*N*_AV_ = 4	*σ*_R_ = 29 (*σ*_T_ = 30)	*σ*_R_ = 76 (*σ*_T_ = 97)

a Results are shown for the two subjects and with different averages.

Signal decay parameters estimated with the NR and MP methods from the averaged 16 (*N*_AV_ = 16) DW images have no actual difference owing to the high SNR and served as reference values. The SNR of nonaveraged DW images at *b* = 1000 s/mm^2^ was higher than 10 for subject 1, and hence the MP and NR algorithms gave identical estimates. For *subject* 2, nonaveraged DW images at *b* = 1000 s/mm^2^ had an SNR of ∼3 owing to the thinner slices.

The median value of the *D*_ME_ across the 16 single measurements for subject 2 is 0.94 × 10^−3^ for the NR, and 1.03 × 10^−3^ mm^2^/s for the MP, respectively (sig., <0.01), whereas the *D*_ME_ from the averaged (*N*_AV_ = 16) DW images is 1.06 × 10^−3^ mm^2^/s. Similarly, the median value of *D*_fast_ is 2.14 × 10^−3^ for NR, 2.22 × 10^−3^ mm^2^/s for MP across the 16 single measurements and 2.4 × 10^−3^ mm^2^/s for the *N*_AV_ = 16. The median value of the heterogeneity index *α* is 0.72 for NR, 0.74 for MP across the 16 single measurements, and 0.75 for the *N*_AV_ = 16. Finally, the perfusion fraction *f* is 0.22 for NR, 0.20 for MP across the 16 single measurements, and 0.19 for the *N*_AV_ = 16.

### Head and Neck DW Images

#### Univariate Analysis

*Benign versus metastatic status*. The median values of signal decay parameters across all patients for benign and metastatic lymph nodes derived by NR and MP algorithms are listed in Table5. As expected from the cellularity, the diffusivity in benign nodes is higher than metastatic nodes 23; hence, *D*_ME_ and *D*_slow_ are significantly higher (MWU sig < 0.01) in the benign nodes for both NR and MP estimates.

**Table 5:** Median Values of All Signal Decay Parameters (Table 2 Along the Benign and the Metastatic Nodes^a^

		*D*_ME_	*D*_slow_	*D*_fast_	*D*_SE_	*α*	*D*_BE_	D^*^_BE_	*f*
NR	Benign	1.14	1.14	2.28	0.96	0.66	0.67	6.45	0.28
	iQR benign	0.26	0.26	1.11	0.27	0.14	0.16	3.37	0.08
	Metastatic	1.02	1.02	1.57	0.86	0.76	0.69	4.19	0.22
	iQR metastatic	0.17	0.17	0.28	0.18	0.09	0.21	2.16	0.03
	MWU sig	0.01	0.01	<0.01	0.73	<0.01	0.42	<0.01	<0.01
MP	Benign	1.21	1.21	2.21	0.9	0.61	0.54	6.25	0.34
	iQR benign	0.29	0.29	1.05	0.2	0.14	0.31	2.51	0.15
	Metastatic	1.02	1.00	1.66	0.86	0.76	0.69	4.31	0.23
	iQR metastatic	0.18	0.18	0.3	0.18	0.09	0.2	2.02	0.03
	MWU sig	<0.01	0.02	0.01	0.21	<0.01	0.06	0.01	<0.01

a Interquartile range and Mann–Whitney *U*-significance test (MWU sig.) are shown to illustrate the distribution of the signal decay parameters along the nodes and whether the median values of benign nodes are significantly different from the ones of metastatic nodes. Results are shown for the estimates from the NR and the MP method. Diffusion coefficients (abbreviations shown in Table1 are given in units of 10^−3^ mm^2^/s.

*D*_fast_, D*_BE_, and *f* were significantly higher, and *a* significantly lower for metastatic than benign lymph nodes (MWU sig., <0.05). All other parameters, whether derived using MP or NR algorithms, were not significantly different (MWU sig., >0.05) between benign and metastatic nodal states. ROC and AUC for univariate prediction of metastatic nodal disease status were greatest for perfusion related parameters (*f*, *a*, and *D*_fast_, Table6.

**Table 6:** Univariate AUCs of the ROC Curves for the Original Data Set of the Signal Decay Parameters that can Significantly (MWU sig., <0.05; Table5 Discriminate Between Benign and Metastatic Nodes*^a^*

				Asymptotic 95% CI	
		AUC	Std.	Lower bound	Upper bound
NR	*D*_ME_	0.75	0.08	0.60	0.91
	*D*_fast_	0.87	0.07	0.74	1.00
	*α*	0.90	0.06	0.78	1.00
	*F*	0.89	0.06	0.77	1.00
	D^*^_BE_	0.78	0.08	0.62	0.94
MP	*D*_ME_	0.78	0.08	0.62	0.93
	*D*_fast_	0.81	0.08	0.65	0.97
	*α*	0.93	0.06	0.82	1.00
	*F*	0.89	0.06	0.77	1.00
	D^*^_BE_	0.74	0.09	0.57	0.91

^a^Abbreviations are shown in Table1. CI is the confidence interval.

Finally, no significant correlation was found (*P* > 0.05) between any of the signal decay parameters and the age of the patients.

**FIG 6:**
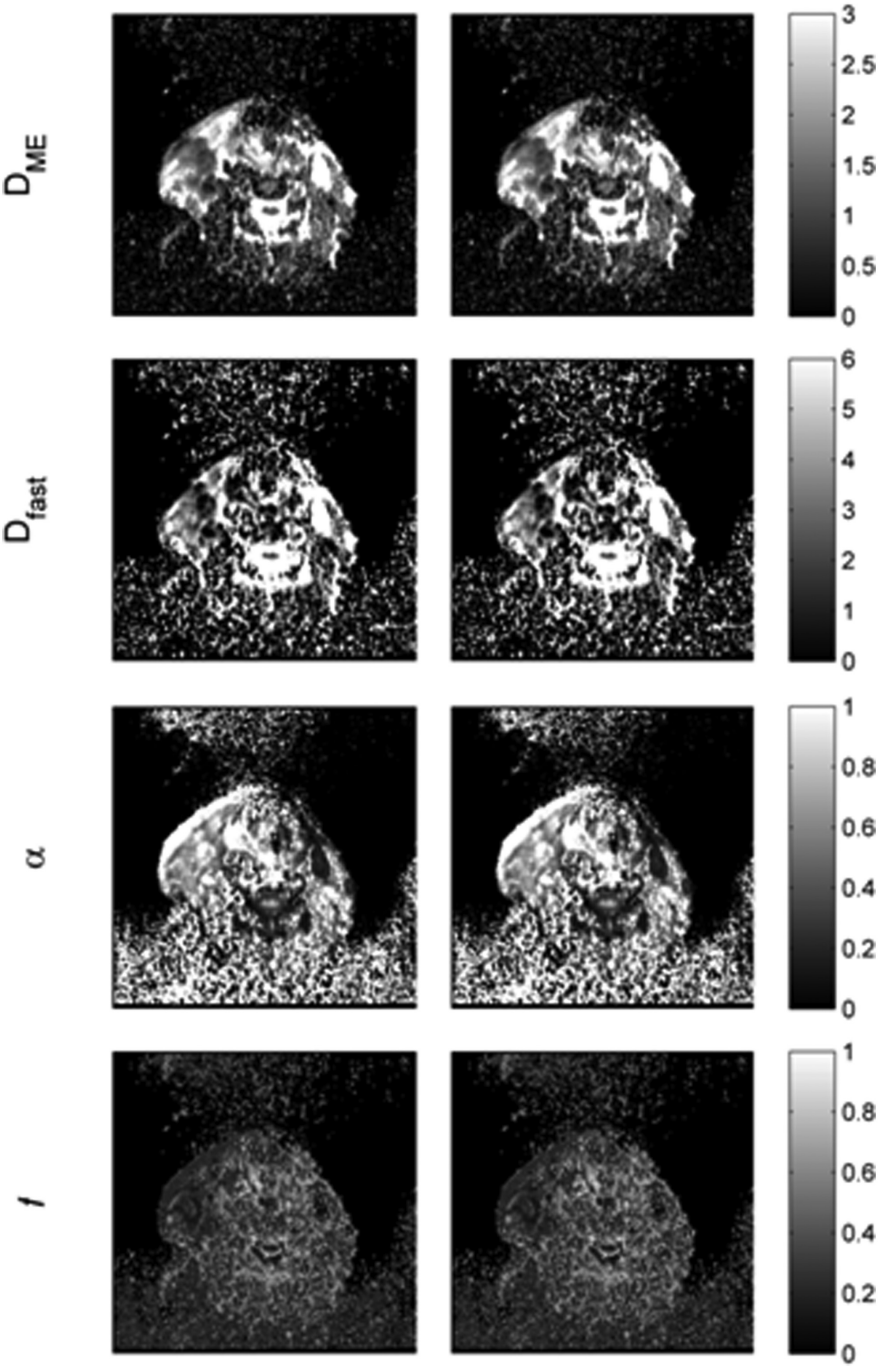
Parametric maps of the *D*_ME_, *D*_fast_, *f*, and *a* diffusion parameters for the NR (left) and MP (right) optimization algorithms (abbreviations are provided in Table1. The maps shown are on the same slice and for the same patient as the DW images in Figure2. Diffusion coefficients *D*_ME_, *D*_fast_ are given in units of 10^−3^ mm^2^/s.

*MP versus conventional NR estimation*. Table5 lists the median values across the 40 patients for each derived signal decay parameter estimated with NR and MP. The signal decay parameters that were significantly different between the MP and NR were *D*_fast_ (W sig. = 0.05), *α* (W sig. = 0.03), and *f* (W sig. = 0.05). *D*_ME_ was not significantly different (W sig. = 0.06). The rest of the signal decay parameters were also not significantly different with W sig. of >0.2. Signal decay parameters estimated with NR were correlated to decay parameters estimated with MP, with a correlation coefficient Kcc close to 1 (Kcc > 0.8). Parametric maps of *D*_ME_, *D*_fast_, *f*, and *a* estimated with NR and MP are shown in Figure6.

### Predictive Model Analysis

Table5 lists the signal decay parameters that could significantly discriminate between benign and metastatic lymph nodes, and a univariate ROC analysis for the parameters is summarized in Table6. Many of the signal decay parameters were correlated, for instance, *f* and *a* have a Kcc of −0.64. Both *f* and *a* are related to perfusion, but *f* closer to 1 indicates high perfusion, whereas *a* close to 1 indicates no perfusion; this explains the negative sign in their Kcc. Similarly, *D*_fast_ and D*_BE_ have an absolute Kcc higher than 0.5 with both *f* and *a*. Diffusion-related parameters *D*_ME_, *D*_SE_, and *D*_BE_ are also correlated with a Kcc of >0.6.

*D*_ME_ and *a* had the highest AUC for the discrimination of benign from metastatic nodes and were weakly correlated Kcc ≤ 0.3. Consequently, the multiparametric logistic regression models were built using *D*_ME_ and *a* decay parameters. Table7 summarizes the performance of the two multiparametric models on the original data set and on independent samples (following leave-one-out and leave-two-out analysis).

**Table 7:** AUCs of the ROC Curves of the Multiparametric Logistic Regression Models Based on Signal Decay Parameters Estimated with NR (mp-DWI NR) and MP (mp-DWI MP)^a^

				Asymptotic 95% CI	
		AUC	Std.	Lower bound	Upper bound
OD	NR	0.92	0.06	0.80	1.00
	MP	0.95	0.06	0.85	1.00
LOO	NR	0.89	0.07	0.76	1.00
	MP	0.92	0.06	0.80	1.00
LTO	NR	0.87	0.10	0.81	0.93
	MP	0.91	0.09	0.87	0.97

a Abbreviations are shown in Table1. Results are shown for the original data set (OD), following leave-one-out (LOO) and leave-two-out (LTO) analysis. CI is the confidence interval.

**FIG 7:**
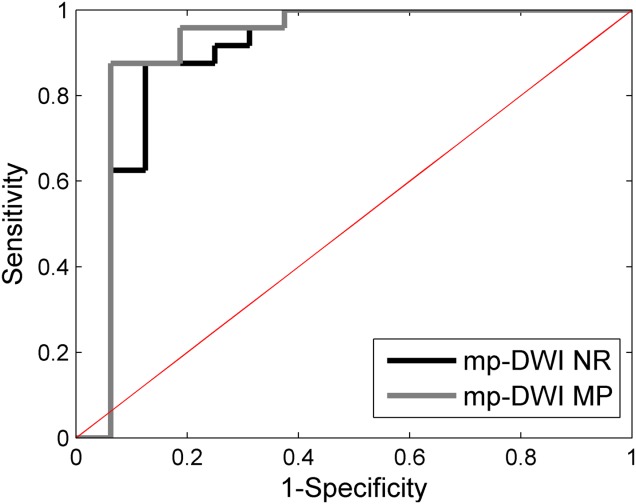
ROC curves for the multiparametric logistic regression models using NR (mp-DWI NR) or MP (mp-DWI MP) signal decay parameter estimates. [Color figure can be viewed in the online issue, which is available at wileyonlinelibrary.com.]

ROC–AUCs for mp-DWI NR/mp-DWI MP on the original data set were 0.92/0.95, respectively. Following leave-one-out analysis, the AUCs slightly dropped to 0.89/0.92, respectively (Fig.7. At 100% sensitivity, both models had 60.0% specificity. At 80% sensitivity, the specificity of mp-DWI NR was 88%, whereas the specificity of mp-DWI MP was 94%. Following leave-two-out analysis, the AUCs dropped to 0.87/0.91, respectively (Table7. At 100% sensitivity, the specificity of mp-DWI NR was 52%, whereas the specificity of mp-DWI MP was 58%. At 80% sensitivity, the specificity of mp-DWI NR was 84%, whereas the specificity of mp-DWI MP was 91%.

## DISCUSSION

Estimation of the underlying noise is important in image processing. Many applications (such as denoising, registration) require knowledge of the statistical properties of the underlying noise of the MR images. DW images in particular have a reduced signal owing to diffusion, the inversion pulse if used for fat suppression and long echo time (owing to the time required for diffusion gradients). If high resolution is needed for mapping of signal decay parameters, the SNR of the DWI is further decreased. Conventional NR algorithms assume normally distributed noise, which can result in biased signal decay parameters especially for low SNR images 5,6).

The focus of this study was to derive and test an accurate noise estimation method, and determine its effect on signal decay parameter estimation and predictive models for the detection of head and neck nodal metastatic disease. In addition to the ME signal decay model, this study also evaluated the effect on parametric estimation for BE and SE models.

### Noise Estimation

The proposed adapted MAD method for noise estimation uses a closed form approximation (Eq. [12] of the noise distribution of averaged data. The suggested closed form approximation accurately fits the convolved Rician distributions for different number of averages and different SNRs. The proposed adapted MAD noise estimator accurately predicted the noise of simulated DWI (***R***^2^ = 0.96) and of the acquired neck DW image at b = 1000 s/mm^2^ for single measurements of different SNRs (*subjects* 1 and 2) and for the averaged (*N*_AV_ = 4) DW image (*subject* 1) of higher SNR. For the averaged DW image of low SNR (*subject* 2), the estimated noise level was ∼20% lower than the noise estimate from the noise-only scan.

### Robust Estimation of Signal-Decay Parameters

Simulation 2 results demonstrated that the advantage of applying the MP method was greatest for low SNR DW images, where NR algorithms underestimated the true *D*_ME_ by up to 40%. The improvements are less apparent for the SE and BE decay models.

Similar results were found for *subject* 2 where MP recovers the *D*_ME_ better than NR (∼9%), whereas no apparent differences were found for the other signal decay parameters. This could be owing to the fact that decay parameters from the SE and BE models are more dependent on the low *b*-value DW images that have higher SNR, whereas decay parameters from the ME model are more dependent on the high *b*-value DW images. Moreover, the SE and BE models have higher degrees of freedom compared to the ME model that makes the fitting to the decay curve less affected by noise.

When parameter thresholds are proposed for the classification of benign/metastatic nodal disease status, the accurate estimation of the parameters is essential. In particular, *D*_ME_ estimates between benign and metastatic nodes derived with NR algorithms overlap, limiting the specificity of absolute *D*_ME_ thresholds 24). We suggest that part of this overlap may be owing to the calculation errors, owing to noise-induced bias, and that MP algorithms may provide better separation and act as more robust classifiers. For our cohort of 40 patients, there was a significant difference of median *D*_ME_ between benign and metastatic nodes for NR and MP (MWU sig = 0.01 and MWU sig., <0.01, respectively). Furthermore, *D*_ME_ was a fair classifier of nodal disease status (ROC–AUC = 0.75/0.78) whether estimated using NR/MP algorithms, respectively.

### Classification of Nodal Disease Status

**FIG 8:**
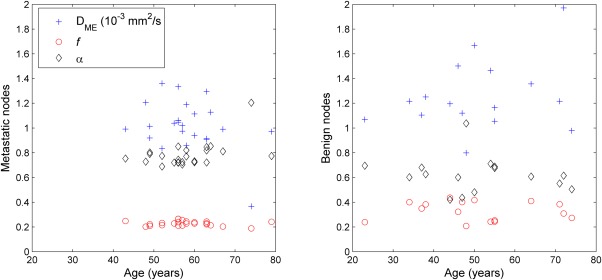
Scatter plot between the diffusion coefficient *D*_ME_ and the heterogeneity index *α* on both patients (red circles) and volunteers (blue crosses) to visualize the classification ability of perfusion versus diffusion-related parameters.

Our univariate analysis indicates that signal decay parameters related to tissue perfusion may be most effective for the classification of nodal disease status. Specifically, the diffusion coefficient *D*_fast_, the perfusion fraction *f*, and the heterogeneity index *α* were the most predictive of nodal disease status. Figure8 shows the classification ability of perfusion (heterogeneity index *α*) versus diffusion (diffusion coefficient *D*_ME_)-related parameters. Metastatic nodal tissue had significantly reduced *D*_fast_ and *f*-values (MWU sig., <0.01) in keeping with reduced perfusion. In contrast, the *α*-value of metastatic nodes was significantly greater than benign nodes (MWU sig., <0.01) which supports less heterogeneity of diffusion rates as would be expected with a reduction in perfusion. Reduced perfusion has been confirmed by Jansen et al. 25 in metastatic cervical nodes in patients with head and neck squamous cell carcinoma.

When comparing MP- and NR-derived parameters, there was no significant difference in the classification performance of the parameters. To avoid overtraining of the derived multiparametric predictive models, only uncorrelated signal decay parameters with significant contribution were included in the final logistic regression diagnostic multiparametric models.

Besides diffusion-weighted studies, classification of nodal status has also been performed with other imaging techniques such as dynamic contrast enhancement MRI, and Fluorodeoxyglucose (^18^F) positron emission tomography. Multiparametric MRI models based on ADC and dynamic contrast enhancement parameters were reported to have ROC–AUC of 0.85 26, and ^18^F positron emission tomography models had ROC–AUC of 0.851 27. The multiparametric predictive model proposed in this study (mp-DWI MP) had an ROC–AUC of 0.95 on the original data set, and 0.91 following leave-two-out analysis (Table7).

## CONCLUSIONS

In summary, the proposed adapted MAD method can predict the noise of DW images and when incorporated into a MP algorithm can correct for the bias in signal decay parameters induced by noise. In the current application, MP algorithms did not significantly improve the classification of benign and metastatic nodal status in a clinical data set, possibly owing to the high SNR (>6, at *b* = 1000 s/mm^2^) of the averaged DW (*N*_AV_ = 4) images. However, the findings from both *simulation* 2 for SNR < 5 and *subject* 2 indicate that for lower SNR, MP-derived decay parameters would be more accurate than NR. Future study could include applications where the SNR is low, for example, in faster scans requiring less averages, or in higher resolution data sets with smaller voxels.
